# Association between pain and sarcopenia in middle-aged and older adults: a systematic review and meta-analysis

**DOI:** 10.3389/fpubh.2026.1891810

**Published:** 2026-08-17

**Authors:** Xiang Li, Yujian Chen, Yannan Zhao, Yao Jiang, Zexing Wen, Wei Xie

**Affiliations:** 1 School of Physical Education and Health, Chengdu University of Traditional Chinese Medicine, Chengdu, China; 2 School of Acupuncture and Tuina, Chengdu University of Traditional Chinese Medicine, Chengdu, China; 3 School of Leisure and Sports, Chengdu Sport University, Chengdu, China

**Keywords:** sarcopenia, pain, middle-aged and older adults, association, meta-analysis

## Abstract

**Background:**

Sarcopenia contributes substantially to functional decline in middle-aged and older adults. Pain is common in this population and may be associated with impaired muscle health; however, the magnitude and clinical characteristics of this association remain uncertain. This systematic review and meta-analysis aimed to quantify the association between pain and sarcopenia-related outcomes in middle-aged and older adults.

**Methods:**

PubMed, Embase, Web of Science, and the Cochrane Library were searched from database inception to April 10, 2026. Observational studies involving adults aged ≥50 years, or populations with a mean age of ≥50 years, that reported the association between pain and sarcopenia-related outcomes were included. Adjusted odds ratios (ORs) and 95% confidence intervals (CIs) were pooled using random-effects models, with between-study variance estimated by the restricted maximum likelihood method. Subgroup and secondary meta-analyses were conducted according to consensus-based sarcopenia criteria, pain severity, and anatomical pain site. Studies using SARC-F were excluded from the corresponding main analyses and were considered only in separate sensitivity analyses. Leave-one-out sensitivity analyses, funnel-plot inspection, and Egger's regression test were also performed. The review was registered in PROSPERO (CRD420261406169).

**Results:**

Nine observational studies were included in the systematic review, of which six studies using consensus-based sarcopenia criteria contributed to the primary meta-analysis. Pain was significantly associated with higher odds of sarcopenia (pooled OR = 1.26, 95% CI: 1.16–1.38; *p* < 0.001), with low between-study heterogeneity (I^2^ = 1.43%). No significant difference was observed between studies using the AWGS 2019 and EWGSOP2 criteria (p for subgroup difference = 0.83). The pooled ORs were 1.33 (95% CI: 1.07–1.65) for mild pain, 1.49 (95% CI: 1.17–1.89) for moderate pain, and 2.17 (95% CI: 1.32–3.57) for severe pain. Back or low-back pain and joint or knee pain were also positively associated with sarcopenia, with pooled ORs of 1.72 (95% CI: 1.28–2.31) and 1.78 (95% CI: 1.12–2.83), respectively. In a sensitivity analysis of the overall pain association, inclusion of PPB 2022, which used SARC-F, increased the pooled OR to 1.56 (95% CI: 1.06–2.31), markedly increased heterogeneity (I^2^ = 94.24%), and reduced result stability. In a separate site-specific sensitivity analysis, inclusion of CLC 2025 yielded a pooled OR of 1.95 (95% CI: 1.30–2.91) for joint or knee pain, and the positive direction of the association was maintained in the corresponding leave-one-out analysis. Egger's test did not indicate statistically significant small-study effects (*p* = 0.533), although its power was limited by the small number of studies.

**Conclusion:**

Pain was associated with higher odds of sarcopenia and sarcopenia-related outcomes in middle-aged and older adults. Positive associations were also observed for severe pain and for site-specific back or low-back pain and joint or knee pain in analyses restricted to studies using consensus-based sarcopenia criteria, although substantial heterogeneity in several secondary analyses warrants cautious interpretation. Sensitivity analyses incorporating SARC-F-based studies yielded positive pooled estimates; however, screening-defined risk of sarcopenia should not be considered equivalent to sarcopenia confirmed using consensus-based criteria. Given the observational nature of the evidence, causal conclusions cannot be drawn. These findings support consideration of sarcopenia screening and physical-function assessment among middle-aged and older adults with persistent or clinically significant pain.

**Systematic Review Registration:**

https://www.crd.york.ac.uk/PROSPERO/view/CRD420261406169, identifier [CRD420261406169].

## 1 Introduction

Sarcopenia is characterized by a progressive decline in skeletal muscle mass, muscle strength, and physical performance and is an important contributor to functional impairment in middle-aged and older adults (1, 2). Pain, including chronic musculoskeletal pain and site-specific pain, is also highly prevalent in later life and is frequently accompanied by restricted mobility, psychological distress, and reduced quality of life (3, 4). The coexistence of pain and sarcopenia may present a particular clinical challenge. Resistance exercise and nutritional interventions are central components of sarcopenia management (5, 6), whereas persistent pain may limit mobility and participation in exercise-based rehabilitation. Clarifying the relationship between these conditions may therefore help identify populations who could benefit from closer assessment of muscle health and physical function (7).

Several biological and behavioral pathways may plausibly contribute to an association between pain and sarcopenia. Chronic or severe pain may promote kinesiophobia and activity avoidance, potentially leading to physical inactivity, muscle disuse, and functional decline (8). Persistent pain may also coexist with systemic low-grade inflammation. Elevated inflammatory markers, including C-reactive protein and interleukin-6, may adversely affect muscle homeostasis by promoting protein catabolism and impairing muscle protein synthesis (9). In addition, persistent pain may be accompanied by sleep disturbance (10). Abnormal sleep duration and depressive symptoms have each been associated with sarcopenia-related outcomes (11, 12). Reduced appetite or inadequate nutritional intake may further contribute to loss of muscle mass and strength (13). These pathways remain biologically plausible explanations rather than established causal mechanisms, and the relationship between pain and sarcopenia may be bidirectional.

Although a growing number of observational studies have examined the relationship between pain and sarcopenia, their findings vary according to study population, pain definition, pain severity, anatomical site, study design, and the method used to assess sarcopenia. In particular, consensus-based diagnostic criteria have been proposed by the Asian Working Group for Sarcopenia and the European Working Group on Sarcopenia in Older People (1, 14). By contrast, SARC-F is a brief screening instrument intended to identify individuals at risk of sarcopenia rather than to provide a confirmatory diagnosis (15, 16). Moreover, the extent to which the association varies across different levels of pain severity and specific anatomical pain sites remains uncertain.

Therefore, this systematic review and meta-analysis aimed to quantitatively synthesize the association between pain and sarcopenia-related outcomes in middle-aged and older adults. We further examined whether the pooled association differed according to consensus-based sarcopenia criteria and conducted secondary analyses according to pain severity and anatomical pain site. Studies using SARC-F were excluded from the main analyses restricted to consensus-based sarcopenia criteria and were considered only in separate sensitivity analyses because SARC-F identifies the risk of sarcopenia rather than providing a confirmatory diagnosis. By clarifying the magnitude and clinical characteristics of the association, this review may inform sarcopenia screening, physical-function assessment, and the design of future prospective studies in individuals with persistent or clinically significant pain.

## 2 Methods

### 2.1 Search strategy

The protocol for this systematic review and meta-analysis was prospectively registered in the International Prospective Register of Systematic Reviews (PROSPERO; registration number: CRD420261406169). This systematic review and meta-analysis was reported in accordance with the Preferred Reporting Items for Systematic Reviews and Meta-Analyses (PRISMA) 2020 statement (17).

Two reviewers (XL and YNZ) independently conducted a systematic search of PubMed, Embase, Web of Science, and the Cochrane Library from database inception to April 10, 2026. The search strategies combined controlled vocabulary terms, including Medical Subject Headings (MeSH) and Emtree terms where applicable, with free-text terms related to pain and sarcopenia. The principal search concepts were “pain” and “sarcopenia,” and the search syntax was adapted to the requirements of each database. The complete line-by-line search strategies and the number of records retrieved from each database are provided in Supplementary Table S1. No restrictions on language or publication type were applied during the database searches.

**Figure 1:**
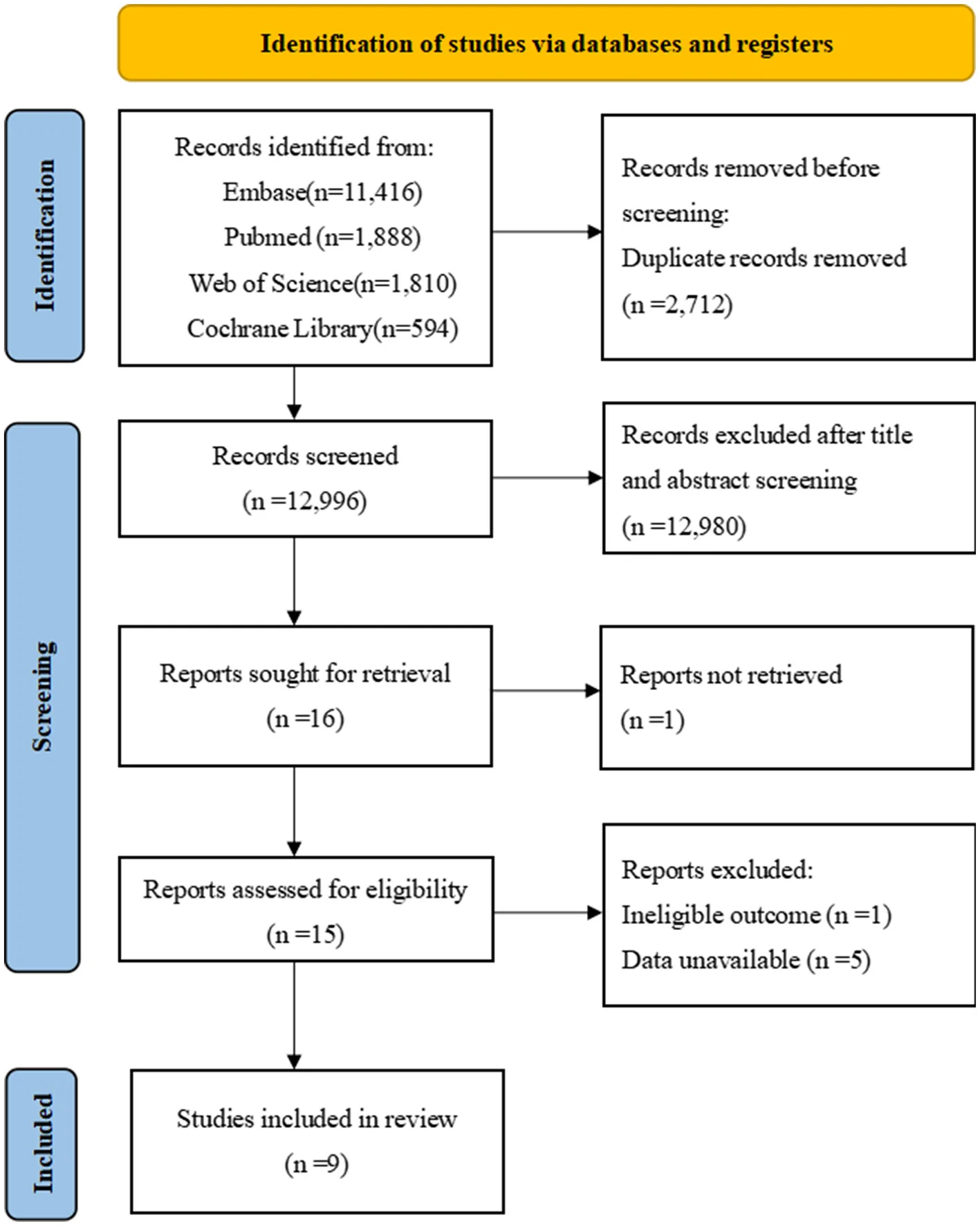
Literature screening flow chart.

In addition, the reference lists of all included studies were manually screened to identify potentially eligible studies that were not retrieved through the electronic database searches. The detailed study identification and selection process is presented in Figure 1.

### 2.2 Inclusion and exclusion criteria

Eligibility criteria were predefined according to the Population, Exposure, Comparator, Outcome, and Study design (PECOS) framework: (1) Population: Studies were eligible if they included middle-aged or older adults aged ≥50 years, or study populations with a mean age of ≥50 years. No restrictions were imposed on sex, ethnicity, or geographic region. Studies involving mixed-age populations were included only when data for participants aged ≥50 years could be separately extracted or when the mean age of the overall sample was ≥50 years. (2) Exposure: The exposure of interest was explicitly assessed pain, including any pain, chronic pain, musculoskeletal pain, widespread or multisite pain, and site-specific pain, such as low back pain or knee pain. In longitudinal studies, pain had to be assessed at baseline, whereas in cross-sectional studies, pain was assessed at the time of the survey. Pain could be ascertained through a clinical diagnosis, a validated pain assessment instrument, such as the Visual Analog Scale, Numeric Rating Scale, or SF-36 Bodily Pain subscale, or a clearly defined questionnaire item. Because the definitions, duration, severity, and anatomical sites of pain varied among the included studies, these characteristics were extracted and examined separately in subgroup or sensitivity analyses where sufficient data were available. (3) Comparator: The comparator consisted of participants from the same source population who were classified as having no pain or who belonged to the study-defined reference category. For analyses of pain severity or number of pain sites, the reference category reported in the original study was retained and recorded. (4) Outcome: Eligible outcomes included the prevalence or incidence of sarcopenia, probable sarcopenia, or risk of sarcopenia. Sarcopenia was assessed using recognized international or regional consensus criteria, including the European Working Group on Sarcopenia in Older People (EWGSOP/EWGSOP2) and the Asian Working Group for Sarcopenia (AWGS/AWGS 2019), or using a validated screening instrument, such as the Strength, Assistance with walking, Rise from a chair, Climb stairs, and Falls questionnaire (SARC-F). Because SARC-F identifies individuals at risk of sarcopenia rather than providing a confirmatory diagnosis, studies using SARC-F were classified separately from studies applying consensus-based criteria. SARC-F-based studies were excluded from the corresponding main meta-analyses and were included only in separate sensitivity analyses. Studies included in the quantitative synthesis were required to report an adjusted odds ratio (OR) with its corresponding 95% confidence interval (CI), or to provide sufficient data from which an OR and 95% CI could be derived. (5) Study design: Eligible studies were original observational studies examining the association between pain and sarcopenia-related outcomes, including prospective or retrospective cohort studies, case-control studies, and cross-sectional studies. Study-design categories were harmonized according to the actual methodological design of each study; therefore, studies described as “follow-up studies” were classified as cohort studies when appropriate.

Studies were excluded if they met any of the following criteria: (1) the study population did not meet the prespecified age criterion and age-specific data could not be extracted; (2) the study exclusively recruited patients with conditions likely to cause substantial muscle wasting independently of pain, such as advanced cancer, cachexia, end-stage organ failure, neuromuscular disease, complete paraplegia, or prolonged immobilization; (3) pain was not assessed as a distinct exposure, or the outcome was not sarcopenia, probable sarcopenia, or risk of sarcopenia; (4) neither an adjusted association estimate with a 95% CI nor sufficient data for its calculation were available; (5) the report was an animal study, case report, review, systematic review, conference abstract, editorial, letter, guideline, protocol, or other non-original publication; (6) the full text or essential data could not be obtained after reasonable attempts to contact the corresponding authors. When multiple publications used overlapping populations, only the publication with the largest sample size, the longest follow-up, or the most comprehensively adjusted analysis was retained for the same quantitative analysis to avoid double-counting participants.

### 2.3 Literature screening

All records retrieved from the electronic database searches were imported into NoteExpress software, and duplicate records were identified and removed. Two reviewers (XL and YNZ) independently conducted the study-selection process in two stages. First, the titles and abstracts of all remaining records were screened against the predefined eligibility criteria. Records considered potentially relevant were retained for full-text assessment. Second, the full texts of potentially eligible reports were retrieved and independently assessed by the same two reviewers to determine their final eligibility.

The reasons for excluding reports during the full-text assessment were recorded and are summarized in the PRISMA 2020 flow diagram. Any disagreements arising at either stage were resolved through discussion and consensus. When consensus could not be reached, a third reviewer (WX) adjudicated the disagreement. The complete study-selection process is presented in Figure 1.

### 2.4 Data extraction

Two reviewers (XL and YNZ) independently extracted data from each eligible study using a standardized data-extraction form. The following information was collected: author and year, country, study design, age, follow-up duration, sarcopenia assessment method and outcome definition, effect estimate with 95% CI, sample size, adjusted covariates, and NOS score.

When an original study reported several multivariable models for the same exposure–outcome association, the effect estimate from the most comprehensively adjusted model was extracted. When a study reported multiple eligible effect estimates according to pain severity, anatomical site, number of pain sites, or other pain characteristics, each estimate was extracted together with its specific exposure definition and comparator category. The potential non-independence of multiple estimates derived from the same study population was recorded, and the rules used to select independent estimates for each quantitative synthesis are described in Section 2.6.

Potentially overlapping study populations were identified by comparing the data source, recruitment period, study setting, participant characteristics, and author group. Any discrepancies in data extraction were resolved through discussion between the two reviewers. When consensus could not be reached, a third reviewer (WX) adjudicated the disagreement.

### 2.5 Quality assessment

The methodological quality of the included studies was assessed according to study design. Cohort studies were evaluated using the original Newcastle–Ottawa Scale (NOS), whereas cross-sectional studies were evaluated using a modified version of the NOS adapted for cross-sectional designs (18).

For cohort studies, the NOS evaluates three domains: selection, comparability, and outcome. The selection domain comprises four items: representativeness of the exposed cohort, selection of the non-exposed cohort, ascertainment of exposure, and demonstration that the outcome of interest was not present at the beginning of the study. The comparability domain evaluates the control of potential confounding factors and permits a maximum of two stars. The outcome domain comprises assessment of the outcome, adequacy of the follow-up duration, and completeness of follow-up. The maximum possible score for cohort studies is nine stars.

For cross-sectional studies, the modified NOS evaluates three domains: selection, comparability, and outcome assessment. The selection domain includes representativeness of the sample, sample-size justification, assessment of non-respondents, and ascertainment of exposure. The comparability domain evaluates whether important confounding factors were controlled for in the study design or statistical analysis. The outcome-assessment domain includes assessment of the outcome and appropriateness of the statistical tests. The maximum possible score for cross-sectional studies is ten stars.

Two reviewers (XL and YNZ) independently assessed each included study. Any disagreements were resolved through discussion and consensus; when consensus could not be reached, a third reviewer (WX) adjudicated the disagreement. Because the original and modified NOS versions have different scoring structures and maximum scores, quality scores were interpreted separately according to study design and were not combined into a single mean score. No universal score threshold was used as an exclusion criterion. The item-level assessments and total scores for cohort and cross-sectional studies are presented separately in Tables 1, 2, respectively.

**Table 1:** Quality assessment of prospective cohort studies using the original NOS.

Study	Items	Scott et al. (28)	Lin et al. (27)	Veronese et al. (25)	Chen et al. (23)	Zhou et al. (22)	Chiew et al. (20)
Selection of population	Representativeness of the exposed cohort	^*^	^*^	^*^	^*^	^*^	^*^
	Selection of the non-exposed cohort	^*^	^*^	^*^	^*^	^*^	^*^
	Ascertainment of exposure	^*^	^*^	^*^	^*^	^*^	^*^
	Demonstration that outcome of interest was not present at start of study	^*^	^*^	^*^	^*^	^*^	/
Comparability of populations	Comparability of cohorts on the basis of the design or analysis	^**^	^**^	^**^	^**^	^**^	^**^
Exposure/outcome assessment	Assessment of outcome	^*^	^*^	^*^	^*^	^*^	/
	Was follow-up long enough for outcomes to occur	^*^	^*^	^*^	^*^	^*^	^*^
	Adequacy of follow-up of cohorts	/	/	/	/	/	/
Score		8	8	8	8	8	6

^*^Indicates that one star was awarded for the corresponding Newcastle-Ottawa Scale item, ^*^^*^indicates that two stars were awarded, and /indicates that no star was awarded for that item.

**Table 2:** Quality assessment of cross-sectional studies using the modified NOS.

Study	Items	Batista et al. (26)	Smith et al. (24)	Kim et al. (21)
Selection of population	Representativeness of the sample	/	^*^	/
	Sample size	/	/	/
	Non-respondents	/	/	/
	Ascertainment of exposure	^*^	^*^	^**^
Comparability of populations	Comparability of subjects on the basis of the design or analysis	^**^	^**^	^**^
Exposure/outcome assessment	Assessment of outcome	^**^	^**^	^**^
	Statistical test	^*^	^*^	^*^
Score		6	7	7

^*^Indicates that one star was awarded for the corresponding Newcastle-Ottawa Scale item, ^**^indicates that two stars were awarded, and /indicates that no star was awarded for that item.

### 2.6 Statistical analysis

Adjusted odds ratios (ORs) and their corresponding 95% confidence intervals (CIs) were used as the effect measures to evaluate the association between pain and sarcopenia-related outcomes. When an individual study reported several multivariable models for the same exposure–outcome association, the effect estimate from the most comprehensively adjusted model was selected. The covariates included in the multivariable model of each study are presented in Table 3.

**Table 3:** Basic characteristics of the included studies.

Study	Country	Study design	Age (years)	Follow-up duration	Sarcopenia assessment method and outcome definition	Effect estimate (95 % CI)	Sample size	Adjusted covariates	NOS score
Scott et al. (28)	Australia	Prospective cohort study	76.5 ± 5.0	5 years	EWGSOP2	OR: Chronic pain 0.78 (0.28–2.17);	1,452	Adjusted for age, BMI, current smoking status, physical activity, number of comorbidities, number of prescription medications and depression symptoms.	8
Batista et al. (26)	Brazil	Cross-sectional study	70 ± 8.14	N/A	SARC-F	OR: pain 4.56 (3.32–6.26)	1,482	Adjusted for age, sex, marital status, education, income, occupation.	6
Lin et al. (27)	China	Prospective cohort study	67.1 ± 4.9	1 year	AWGS 2019	OR: pain 1.83 (1.16–2.89); OR: Mild pain 1.05 (0.43–2.55); OR: Moderate pain 2.30 (1.29–4.11); OR: Severe pain 3.80 (2.12–6.81); OR: Low Back Pain 2.54 (1.52–4.26); OR: Joint pain 2.80 (1.69–4.64);	873	Adjusted for age, sex, BMI, education, marital status, lifestyle (smoking and drinking), depression symptoms, cognitive impairment, anxiety moods, hypertension, diabetes, osteoarthrosis, previous falls, low physical activity and malnutrition.	8
Veronese et al. (25)	England	Prospective cohort study	69.7 ± 7.2	>10 years	EWGSOP2	OR: pain 1.46 (1.18–1.81); OR: Mild pain 1.28 (0.93–1.77); OR: Moderate pain 1.27 (0.84–1.93); OR: Severe pain 1.62 (1.26–2.08); OR: low back pain 1.75 (1.28–2.39); OR: knee pain 1.88 (1.31–2.69);	4,102	Adjusted for age; gender; years of education; body mass categories; ethnicity; marital status; smoking status; Center for Epidemiologic Studies—Depression Scale; physical activity level; number of difficulties in the activities of daily living; presence of multimorbidity; changes in pain during the follow-up.	8
Smith et al. (24)	China, Ghana, India, Mexico, Russia, and South Africa	Cross-sectional study	72.6 ± 11.5	N/A	EWGSOP2	OR: pain 1.22 (1.05–1.42); OR: Mild pain 1.42 (1.04–1.93); OR: Moderate pain 1.43 (1.02–2.00); OR: Severe pain 1.92 (1.09–3.38); OR: back pain 1.26 (1.05–1.51)	14,585	Adjusted for age, sex, education, wealth, chronic conditions, and country	7
Chen et al. (23)	China	Prospective cohort study	59.83 ± 6.60	4 years	AWGS 2019	OR: pain 1.28 (1.01–1.62)	5,568	Adjusted for gender, age, residential area, education, marital status, smoking, drinking, co-morbidities (hyperglycemia, hypertension, stroke, heart disease, chronic lung disease, asthma, liver disease, emotional and mental disorders, and malignancies), and falls.	8
Zhou et al. (22)	China	Prospective cohort study	59.87 ± 9.37	4 years	AWGS 2019	OR: Musculoskeletal pain 1.17 (1.00–1.37); OR: Back pain 1.42 (1.14–1.76); OR: Knee pain 1.23 (1.01–1.50);	8,322	Adjusted for age, gender, residence, marital status, educational level, drinking status, smoking status, hypertension, diabetes mellitus, depressive symptoms, dyslipidemia, cardiovascular disease, pulmonary disease, kidney disease.	8
Kim et al. (21)	Korea	Cross-sectional study	72.69 ± 6.26	N/A	AWGS 2019	OR: Low Back Pain 3.30 (1.60–6.81)	575	Adjusted for body mass index and handgrip strength	7
Chiew et al. (20)	Malaysia	Prospective cohort study	68.21 ± 7.09	5 years	SARC-F	OR: Knee pain 2.72 (1.61–4.59)	577	Adjusted for age, female gender, ethnicity, diabetes mellitus, obesity	6

To avoid double-counting participants and to account for the potential non-independence of multiple estimates from the same study, each independent study population contributed no more than one effect estimate to each meta-analysis. For the primary analysis, the overall effect estimate comparing participants with pain and those without pain was preferentially selected. Effect estimates according to pain severity or anatomical pain site were not included simultaneously in the primary analysis but were used only in the corresponding secondary meta-analyses. Accordingly, different estimates derived from the same study population were not treated as independent studies within the same pooled analysis.

Considering the expected clinical and methodological heterogeneity among the included studies, all pooled analyses were performed using an inverse-variance random-effects model. Between-study variance was estimated using the restricted maximum likelihood (REML) method. Statistical heterogeneity was assessed using Cochran's Q test, the I^2^ statistic, and the between-study variance estimate (τ^2^) (19). A *p*-value of < 0.10 for Cochran's Q test was considered to indicate statistically detectable heterogeneity, whereas I^2^ values were used to describe the magnitude of heterogeneity. All analyses consistently used a random-effects model.

Secondary and subgroup meta-analyses were conducted according to sarcopenia assessment criteria, pain severity, and anatomical pain site when sufficient independent effect estimates were available. For analyses according to sarcopenia assessment criteria, studies using the Asian Working Group for Sarcopenia criteria were analyzed separately from those using the European Working Group on Sarcopenia in Older People criteria. Pain-severity analyses were conducted separately for mild, moderate, and severe pain.

Pain-site analyses were conducted separately for back or low-back pain and joint or knee pain. The main pain-site analyses were restricted to studies using consensus-based sarcopenia criteria. Accordingly, CLC 2025, which assessed the risk of sarcopenia using SARC-F, was excluded from the main joint or knee pain meta-analysis and was included only in an additional sensitivity analysis. A leave-one-out sensitivity analysis was subsequently performed for the expanded four-study dataset. Studies using SARC-F were not combined with consensus-defined sarcopenia outcomes in any main meta-analysis.

The robustness of the primary pooled estimate and the expanded sensitivity-analysis datasets was examined using leave-one-out analyses, in which each study was sequentially omitted and the corresponding meta-analysis was repeated. Small-study effects and potential publication bias were explored through visual inspection of funnel-plot symmetry and Egger's regression test. Because fewer than 10 independent studies were included in the primary meta-analysis, the funnel plot and Egger's test were considered to have limited statistical power and were therefore interpreted cautiously.

All statistical tests were two-sided. A *p* value of < 0.05 was considered statistically significant, except for Cochran's Q test, for which a threshold of *p* < 0.10 was used. All statistical analyses were conducted using Stata software, version 17.0.

## 3 Results

### 3.1 Literature screening process and results

The systematic search identified a total of 15,708 records from four electronic databases: Embase (*n* = 11,416), PubMed (*n* = 1,888), Web of Science (*n* = 1,810), and the Cochrane Library (*n* = 594). After the removal of 2,712 duplicate records, 12,996 unique records underwent title and abstract screening. Of these, 12,980 records were excluded because they did not meet the predefined eligibility criteria, and 16 reports were sought for retrieval.

One report could not be retrieved, leaving 15 reports for full-text eligibility assessment. Following full-text assessment, six reports were excluded because the outcome did not meet the eligibility criteria (*n* = 1) or the data required for the review were unavailable (*n* = 5). Consequently, nine studies were included in the systematic review (20–28). The number of studies contributing to the individual quantitative syntheses varied according to the availability of eligible independent effect estimates. The complete study-selection process is presented in the PRISMA 2020 flow diagram in Figure 1.

### 3.2 Basic characteristics of the included literature

Table 3 summarizes the characteristics of the nine observational studies included in this systematic review. Three studies were conducted exclusively in China (22, 23, 27), whereas the remaining six were conducted in Australia, Brazil, England, Korea, Malaysia, or multinational populations from China, Ghana, India, Mexico, Russia, and South Africa (20, 21, 24–26, 28). Regarding study design, six studies were prospective cohort studies (20, 22, 23, 25, 27, 28) and three were cross-sectional studies (21, 24, 26). Studies described as follow-up studies were classified as cohort studies according to their actual methodological design.

The nine studies included a total of 37,536 participants, with individual sample sizes ranging from 575 to 14,585. All studies involved middle-aged or older populations, and the reported mean ages ranged from 59.83 to 76.50 years. Among the cohort studies, follow-up durations ranged from 1 year to more than 10 years.

Sarcopenia-related outcomes were assessed using the Asian Working Group for Sarcopenia 2019 criteria in four studies, the European Working Group on Sarcopenia in Older People 2 criteria in three studies, and the SARC-F screening instrument in two studies. Studies using SARC-F evaluated the risk of sarcopenia rather than a confirmatory diagnosis. Pain exposure was heterogeneous and included any pain, chronic pain, musculoskeletal pain, back or low-back pain, joint or knee pain, and different levels of pain severity. The two studies using SARC-F were retained in the systematic review but were excluded from the corresponding main meta-analyses and were considered only in separate sensitivity analyses.

All included studies reported adjusted odds ratios with corresponding 95% confidence intervals. However, the extent of covariate adjustment differed substantially across studies, ranging from models including two covariates to models including approximately 16 covariates. The adjusted variables included demographic characteristics, lifestyle factors, comorbidities, psychological factors, physical activity, nutritional status, and functional limitations. Detailed characteristics of the included studies are presented in Table 3.

### 3.3 Quality assessment

The methodological quality of the included studies was evaluated separately according to study design. Among the six prospective cohort studies assessed using the original Newcastle–Ottawa Scale, the scores ranged from 6 to 8 out of a maximum of 9 stars. Five studies received 8 stars, whereas one study received 6 stars. The cohort studies generally performed well in the selection of participants, ascertainment of pain exposure, control of potential confounding factors, and adequacy of follow-up duration. However, the completeness of follow-up was insufficiently reported in all cohort studies. In addition, one study did not clearly demonstrate that the outcome was absent at baseline and had limitations in outcome assessment.

Among the three cross-sectional studies assessed using the modified Newcastle–Ottawa Scale, the scores ranged from 6 to 7 out of a maximum of 10 stars. One study received 6 stars, and two studies received 7 stars. All three studies received full scores for comparability, outcome assessment, and the appropriateness of statistical analyses. The most common methodological limitations were the absence of sample-size justification and insufficient information regarding non-respondents. Sample representativeness was also limited in two studies.

Because the original and modified versions of the Newcastle–Ottawa Scale have different scoring structures and maximum scores, an overall mean score was not calculated, and the results were interpreted separately according to study design. Detailed item-level assessments and total scores for the cohort and cross-sectional studies are presented in Tables 1, 2, respectively.

### 3.4 Meta-analysis

#### 3.4.1 Association between pain and sarcopenia in middle-aged and older adults

**Figure 2:**
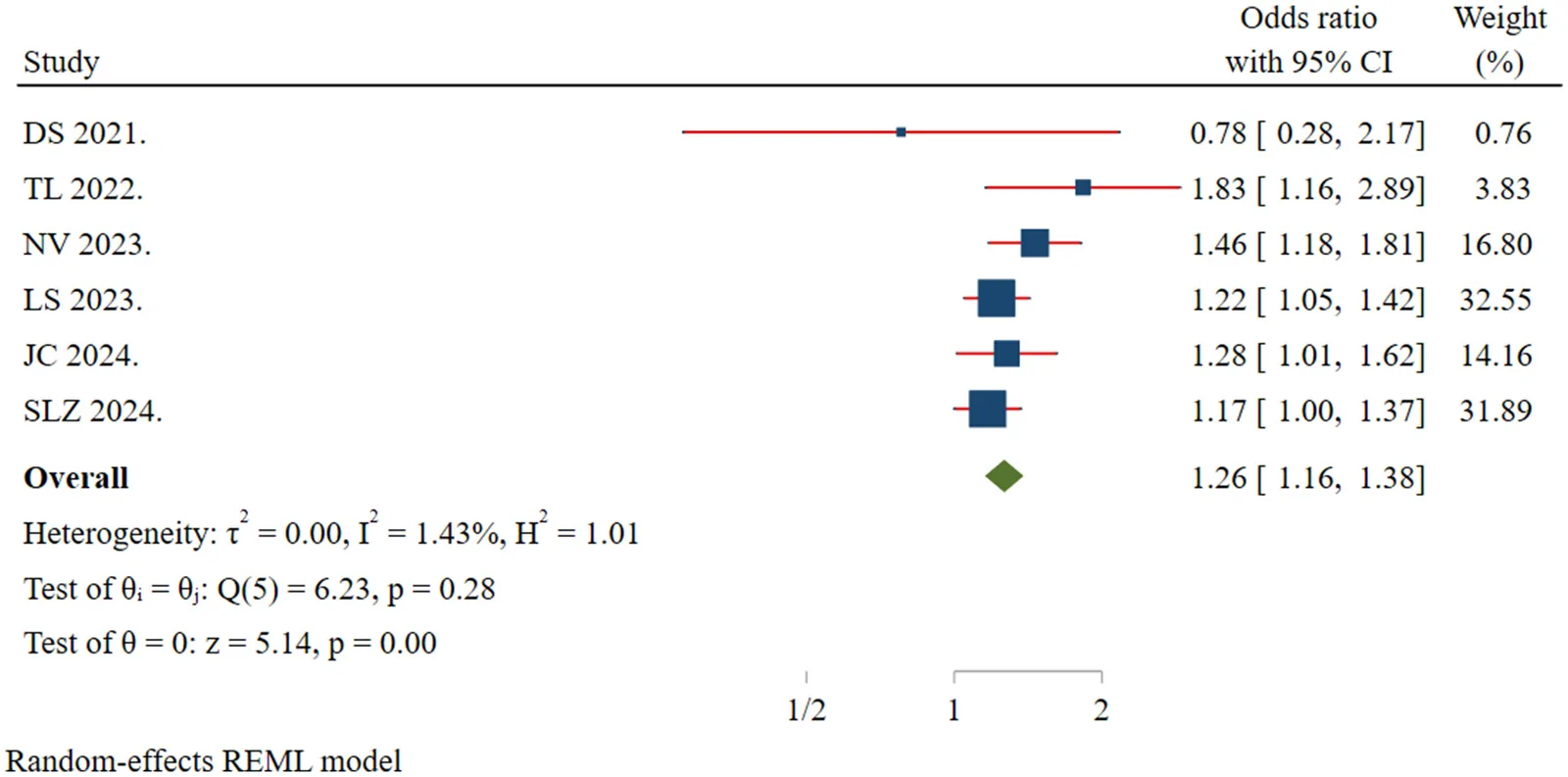
Forest plot of the association between pain and sarcopenia in middle-aged and older adults.

As shown in Figure 2, six studies providing independent overall effect estimates and assessing sarcopenia using consensus-based criteria were included in the primary meta-analysis. Using a random-effects model with between-study variance estimated by the restricted maximum likelihood method, pain was significantly associated with higher odds of sarcopenia in middle-aged and older adults (pooled OR = 1.26, 95% CI: 1.16–1.38; *p* < 0.001). Between-study heterogeneity was low and not statistically significant (I^2^ = 1.43%, τ^2^ = 0.00; Cochran's Q = 6.23, *p* = 0.28). Participants reporting pain therefore had 26% higher odds of sarcopenia than those without pain.

**Figure 3:**
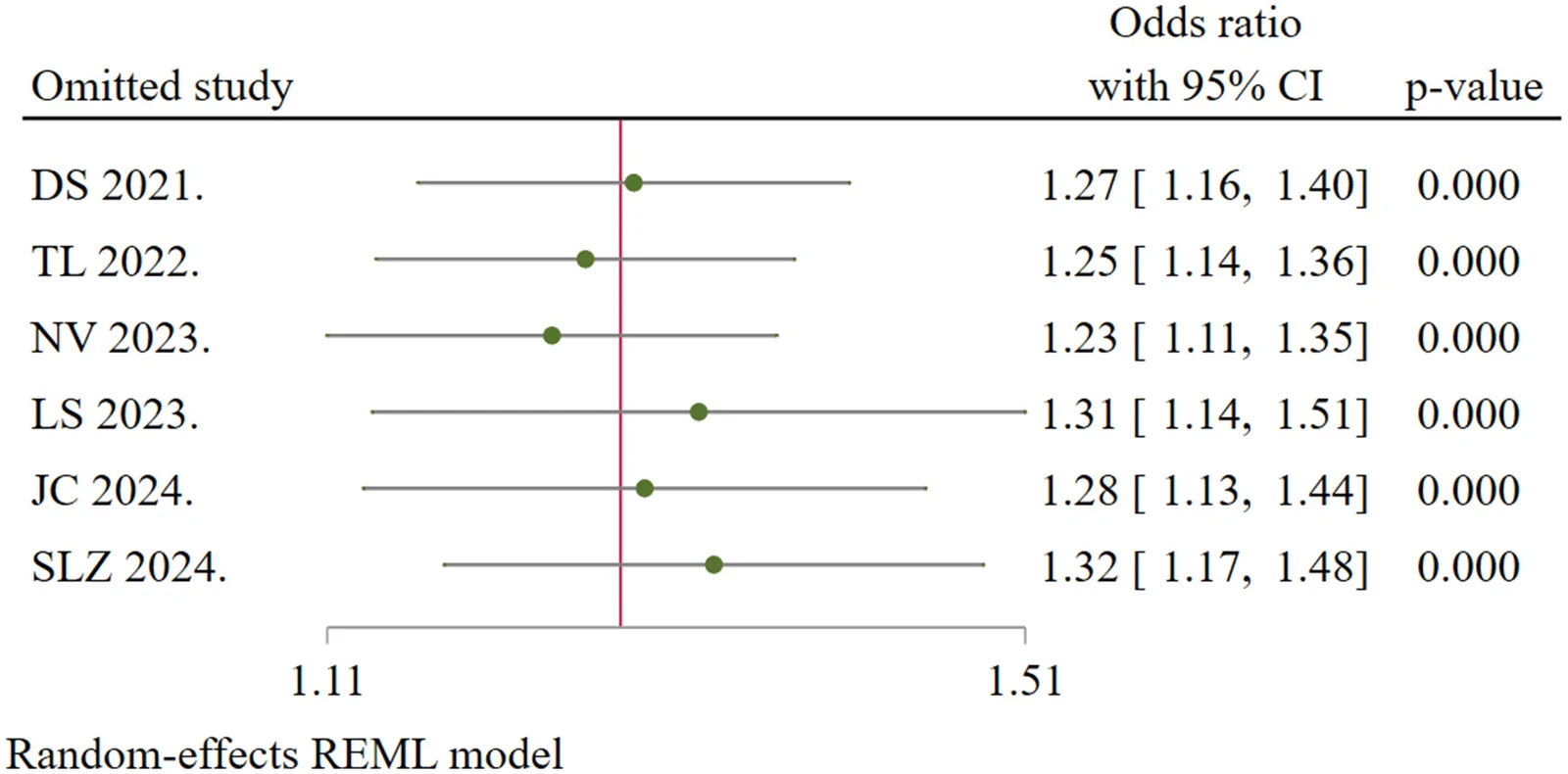
Sensitivity analysis of the association between pain and sarcopenia in middle-aged and older adults.

The robustness of the primary result was examined using a leave-one-out sensitivity analysis (Figure 3). After the sequential omission of each individual study, the recalculated pooled ORs ranged from 1.23 to 1.32, and all corresponding 95% CIs remained above the null value of 1.0. The smallest pooled estimate was observed after the exclusion of NV 2023 (OR = 1.23, 95% CI: 1.11–1.35), whereas the largest was observed after the exclusion of SLZ 2024 (OR = 1.32, 95% CI: 1.17–1.48). These findings support the stability of the primary association and indicate that it was not disproportionately influenced by any single study.

**Figure 4:**
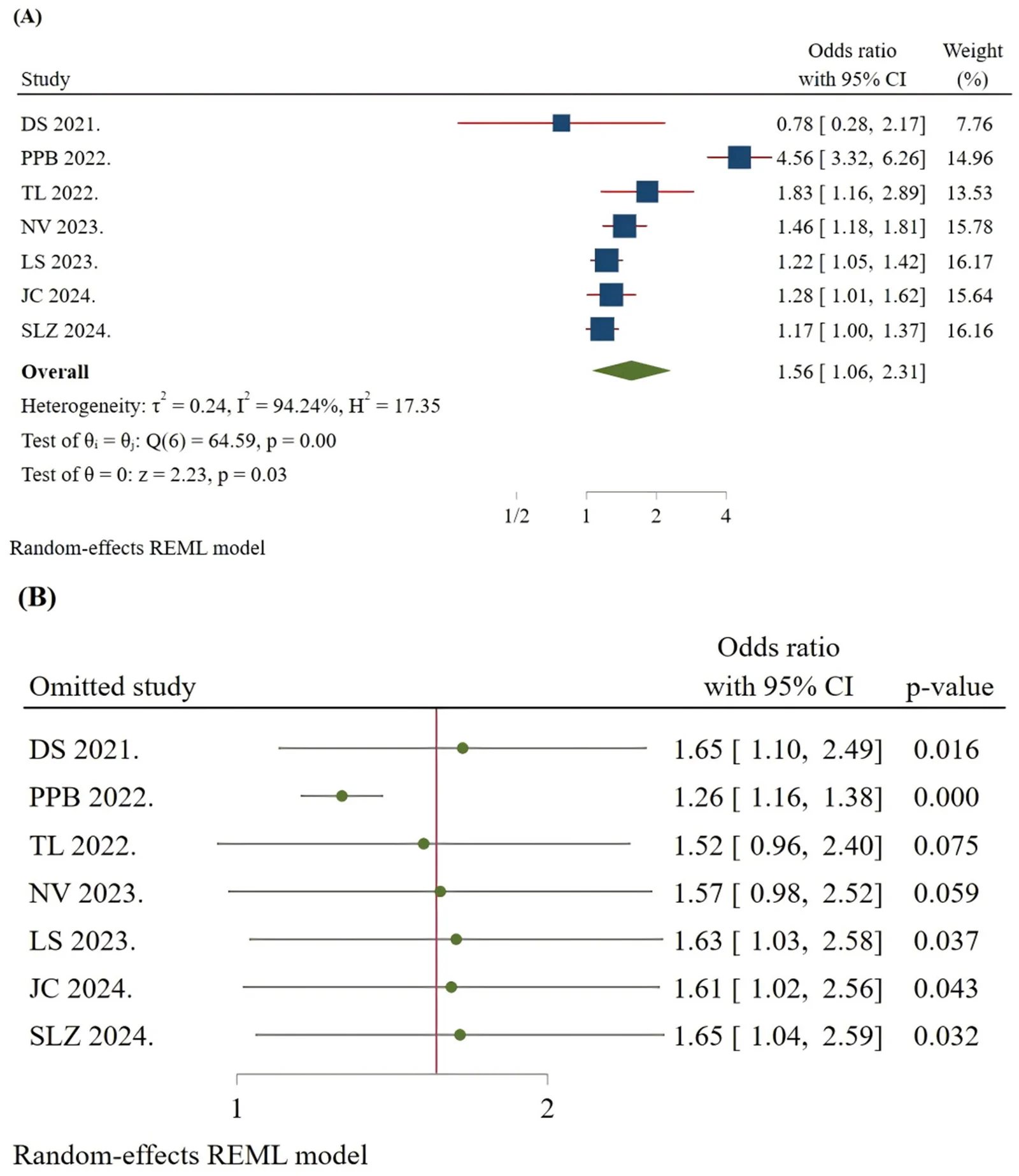
Sensitivity analyses after the additional inclusion of PPB 2022, which assessed the risk of sarcopenia using SARC-F. **(A)** Random-effects meta-analysis after including PPB 2022. **(B)** Leave-one-out sensitivity analysis after including PPB 2022.

An additional sensitivity analysis was conducted by including PPB 2022, which used SARC-F to assess the risk of sarcopenia (Figure 4A). After the inclusion of PPB 2022, the pooled association remained statistically significant (OR = 1.56, 95% CI: 1.06–2.31; *p* = 0.03). However, between-study heterogeneity increased substantially (I^2^ = 94.24%, τ^2^ = 0.24; Cochran's Q = 64.59, *p* < 0.001). In the corresponding leave-one-out analysis (Figure 4B), the direction of the pooled point estimates remained positive after the sequential omission of each study. Nevertheless, statistical significance was not maintained after the exclusion of TL 2022 (OR = 1.52, 95% CI: 0.96–2.40; *p* = 0.075) or NV 2023 (OR = 1.57, 95% CI: 0.98–2.52; *p* = 0.059). When PPB 2022 was excluded, the pooled estimate returned to that of the primary analysis (OR = 1.26, 95% CI: 1.16–1.38). These findings indicate that the expanded analysis incorporating the SARC-F-based outcome was more heterogeneous and less stable than the primary analysis restricted to consensus-based sarcopenia criteria.

#### 3.4.2 Subgroup analyses

**Figure 5:**
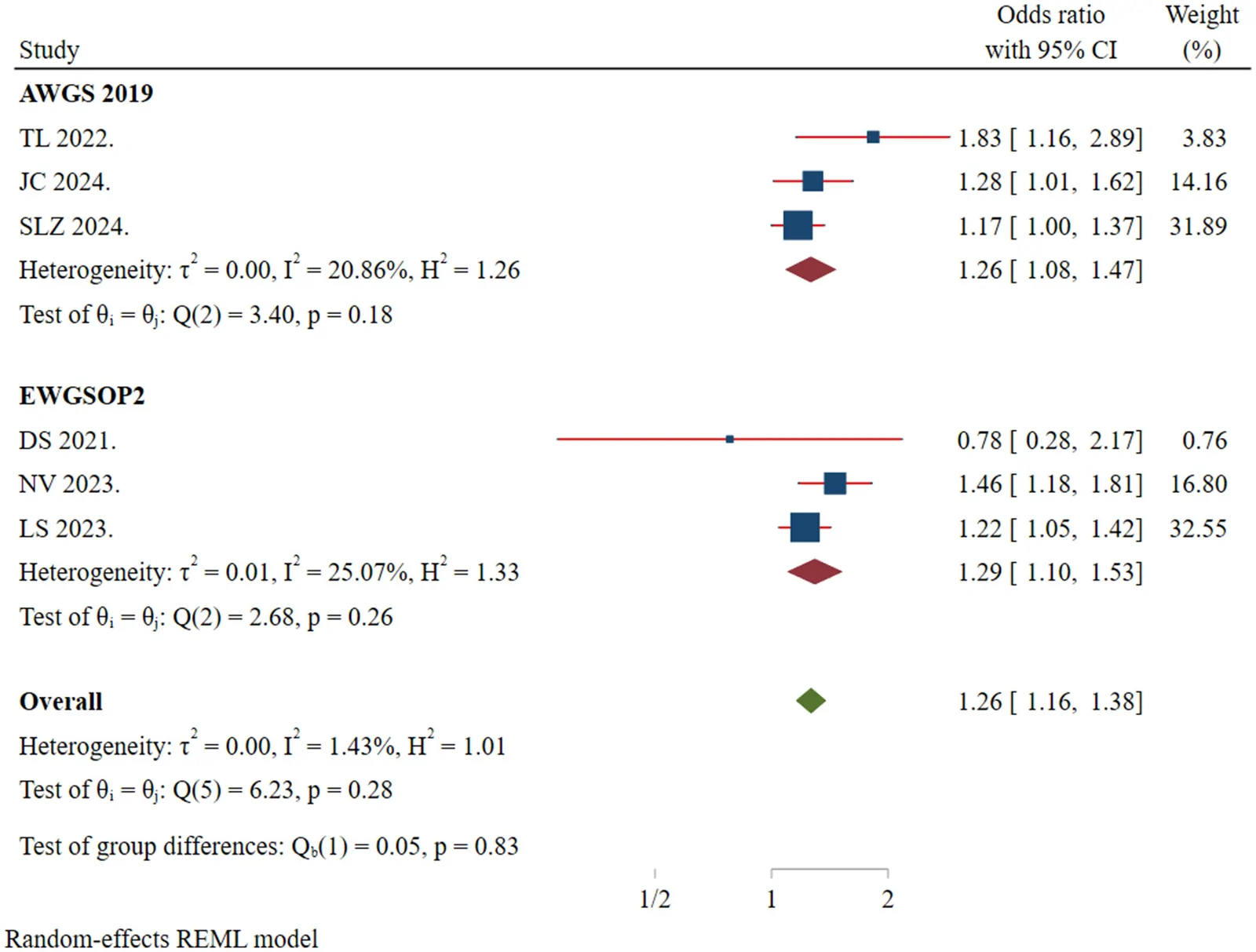
Subgroup analysis according to consensus-based sarcopenia criteria.

To examine whether the primary association differed according to the consensus-based criteria used to assess sarcopenia, studies were stratified into the Asian Working Group for Sarcopenia 2019 (AWGS 2019) and European Working Group on Sarcopenia in Older People 2 (EWGSOP2) groups (Figure 5). Among studies using the AWGS 2019 criteria, pain was significantly associated with higher odds of sarcopenia (pooled OR = 1.26, 95% CI: 1.08–1.47), with low between-study heterogeneity (I^2^ = 20.86%, τ^2^ = 0.00; Cochran's Q = 3.40, *p* = 0.18). A similar association was observed among studies using the EWGSOP2 criteria (pooled OR = 1.29, 95% CI: 1.10–1.53), with low heterogeneity (I^2^ = 25.07%, τ^2^ = 0.01; Cochran's Q = 2.68, *p* = 0.26). The test for subgroup differences was not statistically significant (Qb = 0.05, *p* = 0.83), indicating no evidence that the pooled association differed between the two consensus-based criteria.

#### 3.4.3 Secondary meta-analyses

**Figure 6:**
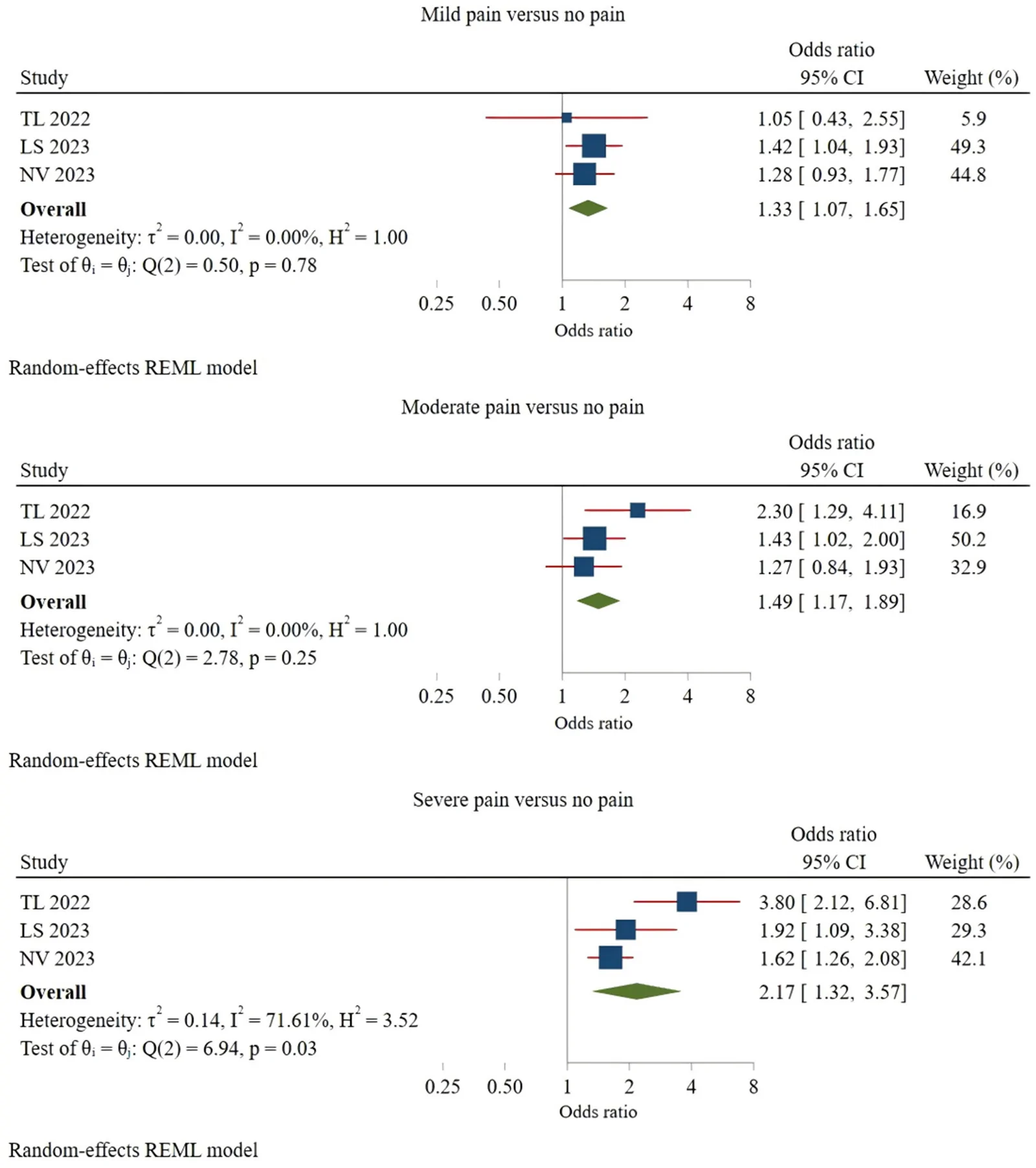
Secondary meta-analyses according to pain severity.

Secondary meta-analyses were conducted separately for mild, moderate, and severe pain compared with no pain (Figure 6). Mild pain was significantly associated with higher odds of sarcopenia (pooled OR = 1.33, 95% CI: 1.07–1.65), with no observed heterogeneity (I^2^ = 0.00%, τ^2^ = 0.00; Cochran's Q = 0.50, *p* = 0.78). The pooled OR for moderate pain was 1.49 (95% CI: 1.17–1.89), also with no observed heterogeneity (I^2^ = 0.00%, τ^2^ = 0.00; Cochran's Q = 2.78, *p* = 0.25). Severe pain was associated with a larger pooled effect estimate (OR = 2.17, 95% CI: 1.32–3.57), although substantial heterogeneity was observed (I^2^ = 71.61%, τ^2^ = 0.14; Cochran's Q = 6.94, *p* = 0.03). Although the pooled point estimates increased across the mild, moderate, and severe pain categories, these categories were examined in separate secondary meta-analyses, and no formal test for trend or between-category differences was performed. The apparent severity gradient should therefore be interpreted cautiously rather than as definitive evidence of a dose–response relationship.

**Figure 7:**
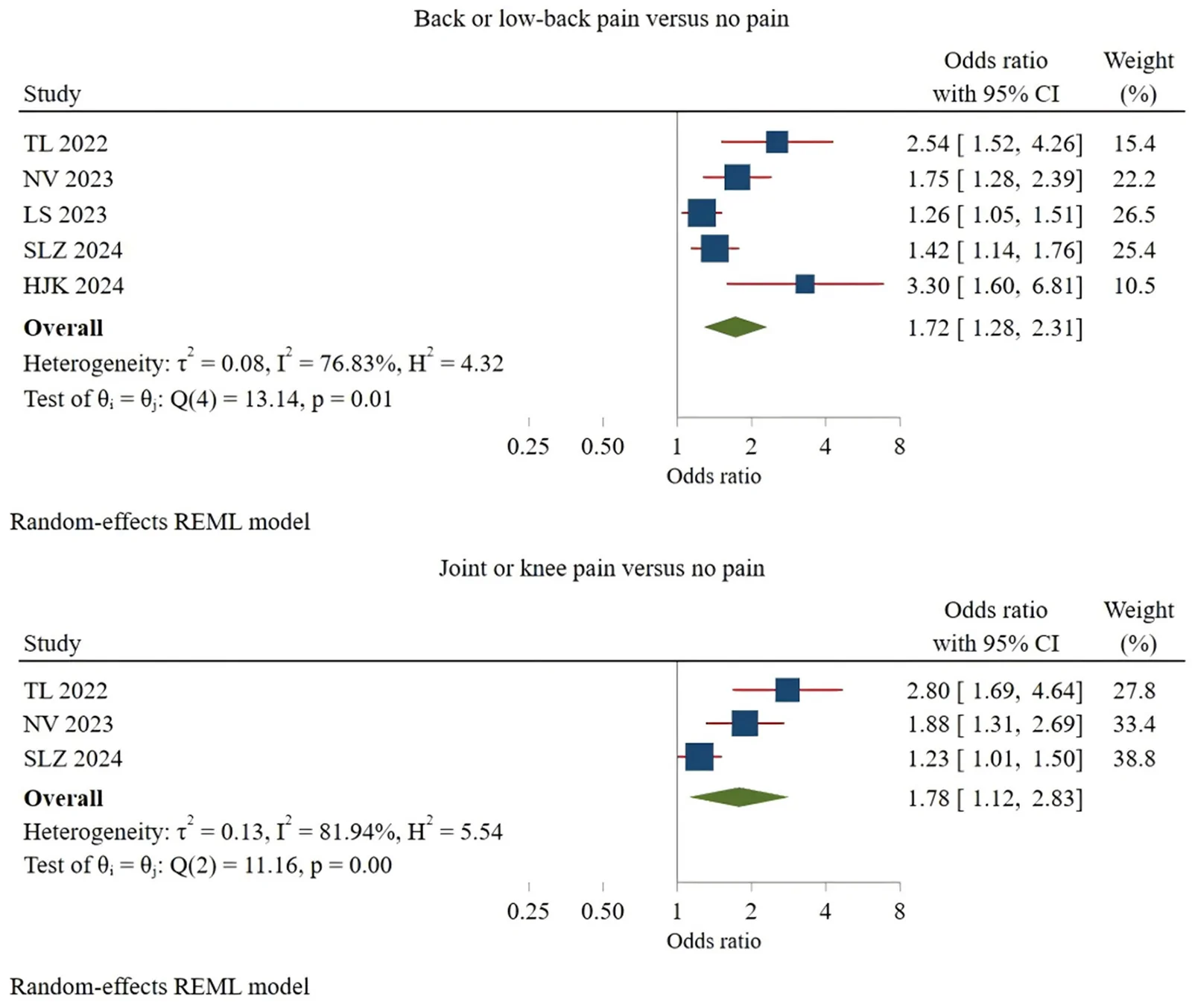
Secondary meta-analyses according to pain site.

Site-specific secondary meta-analyses restricted to studies using consensus-based sarcopenia criteria were performed for back or low-back pain and joint or knee pain (Figure 7). Among five studies, back or low-back pain was significantly associated with higher odds of sarcopenia (pooled OR = 1.72, 95% CI: 1.28–2.31). Substantial between-study heterogeneity was observed (I^2^ = 76.83%, τ^2^ = 0.08; Cochran's Q = 13.14, *p* = 0.011). Among three studies using consensus-based sarcopenia criteria, joint or knee pain was also significantly associated with higher odds of sarcopenia (pooled OR = 1.78, 95% CI: 1.12–2.83). Substantial heterogeneity was observed across studies (I^2^ = 81.94%, τ^2^ = 0.13; Cochran's Q = 11.16, *p* = 0.004). These findings indicate positive associations for both categories of site-specific pain, although the substantial heterogeneity warrants cautious interpretation.

In an additional site-specific sensitivity analysis, CLC 2025, which used SARC-F to identify the risk of sarcopenia, was added to the three studies using consensus-based sarcopenia criteria (Supplementary Figure S1A). The expanded analysis remained statistically significant (pooled OR = 1.95, 95% CI: 1.30–2.91; *p* = 0.001), with substantial between-study heterogeneity (I^2^ = 78.68%, τ^2^ = 0.13; Cochran's Q = 16.04, *p* = 0.001). In the corresponding leave-one-out sensitivity analysis, the pooled ORs ranged from 1.76 to 2.30, and all corresponding 95% CIs remained above 1.0 (Supplementary Figure S1B). When CLC 2025 was omitted, the pooled estimate returned to that of the main consensus-based joint or knee pain analysis (OR = 1.78, 95% CI: 1.12–2.83). These findings indicate that inclusion of the SARC-F-based study did not change the direction or statistical significance of the association. However, because the expanded dataset combined consensus-defined sarcopenia with screening-defined risk of sarcopenia, this result was interpreted separately from the main site-specific analysis and was considered an analysis of sarcopenia-related outcomes rather than confirmed sarcopenia alone.

#### 3.4.4 Publication bias

**Figure 8:**
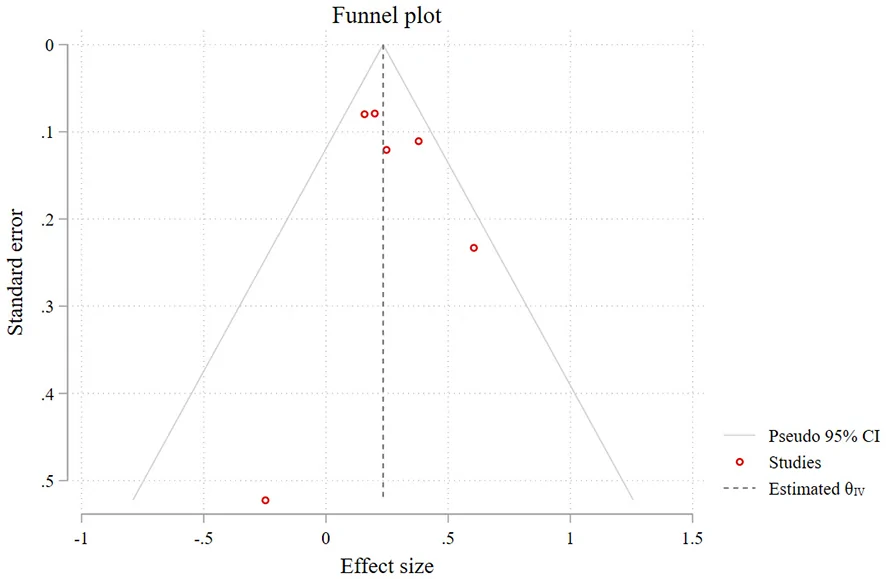
Funnel plot for publication bias.

Potential small-study effects and publication bias in the primary meta-analysis were explored through visual inspection of the funnel plot and Egger's regression test. Visual inspection of the funnel plot did not reveal marked asymmetry (Figure 8). Consistently, Egger's regression test did not provide statistically significant evidence of small-study effects (*z* = 0.62, *p* = 0.533). However, because the primary meta-analysis included only six independent studies, both the visual assessment of funnel-plot symmetry and Egger's regression test had limited statistical power. These findings should therefore be interpreted cautiously, and the possibility of publication bias cannot be excluded.

## 4 Discussion

This systematic review and meta-analysis provides an updated quantitative synthesis of the association between pain and sarcopenia-related outcomes in middle-aged and older adults. Nine observational studies were included in the systematic review, of which six studies providing independent overall effect estimates and using consensus-based sarcopenia criteria contributed to the primary meta-analysis. The primary analysis showed that pain was significantly associated with higher odds of sarcopenia (pooled OR = 1.26, 95% CI: 1.16–1.38). Between-study heterogeneity was low (*I*^2^ = 1.43%), and the leave-one-out analysis produced pooled ORs ranging from 1.23 to 1.32, with all 95% confidence intervals remaining above 1.0. These findings support the stability of the primary association, while the observational nature of the included evidence precludes causal interpretation.

The association was similar across the two consensus-based sarcopenia criteria. The pooled OR was 1.26 (95% CI: 1.08–1.47) among studies using the AWGS 2019 criteria and 1.29 (95% CI: 1.10–1.53) among those using the EWGSOP2 criteria, with no statistically significant difference between the subgroups (*p* = 0.83). This consistency suggests that the observed association was not substantially dependent on the specific consensus-based criteria used to define sarcopenia. However, this finding should not be interpreted as evidence that all sarcopenia assessment methods are interchangeable. When PPB 2022, which used SARC-F to identify the risk of sarcopenia, was added in an additional sensitivity analysis, the pooled OR increased to 1.56 (95% CI: 1.06–2.31), while heterogeneity increased markedly to 94.24%. Moreover, statistical significance was not maintained after the omission of TL 2022 or NV 2023. These findings indicate that the expanded analysis incorporating a screening-based outcome was less stable and that differences in sarcopenia ascertainment may materially affect the pooled estimate. They also support the use of consensus-based studies as the primary analytical set and the separate consideration of studies using SARC-F.

The secondary analyses according to pain severity showed that mild, moderate, and severe pain were each positively associated with sarcopenia. The pooled ORs were 1.33 (95% CI: 1.07–1.65) for mild pain, 1.49 (95% CI: 1.17–1.89) for moderate pain, and 2.17 (95% CI: 1.32–3.57) for severe pain. The progressive increase in the pooled point estimates is suggestive of a severity-related gradient. Nevertheless, these estimates were obtained from separate secondary meta-analyses, and no formal test for trend or between-category interaction was performed. Therefore, the findings should not be regarded as definitive evidence of a dose–response relationship. In addition, substantial heterogeneity was observed in the severe-pain analysis, further warranting cautious interpretation.

Site-specific analyses restricted to studies using consensus-based sarcopenia criteria also showed positive associations. Back or low-back pain was associated with higher odds of sarcopenia (OR = 1.72, 95% CI: 1.28–2.31), and joint or knee pain was similarly associated with higher odds of sarcopenia (OR = 1.78, 95% CI: 1.12–2.83). Both analyses exhibited substantial heterogeneity. This heterogeneity may reflect differences in pain definitions, chronicity, severity, study populations, pain-assessment methods, and covariates included in the adjusted models. When CLC 2025, which used SARC-F, was added in a separate sensitivity analysis, the pooled OR for joint or knee pain increased numerically to 1.95 (95% CI: 1.30–2.91). All pooled estimates remained statistically significant in the corresponding leave-one-out analysis. However, this expanded estimate combined consensus-confirmed sarcopenia with SARC-F-defined risk of sarcopenia and should therefore be interpreted as an association with sarcopenia-related outcomes rather than with confirmed sarcopenia alone. Importantly, the present analyses evaluated specific anatomical categories rather than the number of painful sites. Consequently, the current findings do not support conclusions regarding single-site vs. multisite pain.

Several biological and behavioral pathways may plausibly contribute to the observed association. Chronic or severe pain may promote fear of movement and activity avoidance, potentially resulting in reduced physical activity, muscle disuse, and progressive functional decline (29). Persistent pain may also coexist with systemic low-grade inflammation; inflammatory mediators such as C-reactive protein and interleukin-6 may contribute to impaired muscle protein synthesis and increased protein catabolism (9). In addition, pain may be accompanied by depressive symptoms, sleep disturbance, reduced appetite, or inadequate nutritional intake, which could further adversely affect muscle mass and function (30). These mechanisms remain plausible explanations rather than established causal pathways, and the relationship may also be bidirectional because reduced muscle strength and impaired physical function could increase vulnerability to musculoskeletal pain.

This review has several strengths. The review was prospectively registered and reported in accordance with the PRISMA 2020 statement. A comprehensive search of four electronic databases was performed, and study selection, data extraction, and methodological quality assessment were conducted independently by two reviewers. To address the unit-of-analysis issue, each independent study population contributed no more than one estimate to each quantitative synthesis, while severity-specific and site-specific estimates were restricted to their corresponding secondary analyses. Effect estimates from the most comprehensively adjusted models were extracted, and studies using consensus-based sarcopenia criteria were distinguished from those using SARC-F. The influence of screening-based outcome ascertainment was evaluated through separate sensitivity analyses involving PPB 2022 for the overall pain association and CLC 2025 for the joint or knee pain association, together with corresponding leave-one-out analyses.

Several limitations should also be acknowledged. First, all included studies were observational, and three were cross-sectional. Thus, temporal direction and causality cannot be established, and reverse causation remains possible. Second, only six independent studies contributed to the primary meta-analysis. The relatively small number of studies limited the precision of subgroup and sensitivity analyses and substantially reduced the power of the funnel plot and Egger's regression test. Although Egger's test did not show statistically significant evidence of small-study effects, publication bias cannot be excluded. Third, the definitions and assessment of pain differed considerably across studies, including any pain, chronic pain, musculoskeletal pain, low-back pain, knee or joint pain, and varying severity categories. This clinical heterogeneity limits the direct comparability of the included studies.

Fourth, sarcopenia-related outcomes were evaluated using both consensus-based criteria and SARC-F. Because SARC-F is a screening instrument rather than a confirmatory diagnostic method, studies using SARC-F may represent clinically different outcomes. The marked increase in heterogeneity after inclusion of PPB 2022 in the overall pain analysis illustrates the potential influence of outcome ascertainment. In the joint or knee pain analysis, inclusion of CLC 2025 did not change the direction or statistical significance of the association, but the expanded estimate combined screening-defined risk with consensus-confirmed sarcopenia. Moreover, because only one SARC-F-based study was available for joint or knee pain, a separate pooled SARC-F subgroup and a formal comparison between outcome-assessment methods could not be performed.

Fifth, although adjusted ORs were used, covariate adjustment differed substantially across studies, ranging from relatively limited models to models including approximately 16 demographic, behavioral, psychological, clinical, and functional variables. Residual confounding therefore remains possible, and the pooled estimates should not be interpreted as representing an independent causal effect of pain.

Overall, the available evidence indicates that pain is associated with higher odds of sarcopenia-related outcomes in middle-aged and older adults. Positive associations were observed for severe pain and for site-specific back or low-back pain and joint or knee pain in analyses restricted to consensus-based sarcopenia criteria; however, the limited number of studies and substantial heterogeneity in several secondary analyses warrant cautious interpretation. These findings support consideration of sarcopenia screening and physical-function assessment in middle-aged and older patients presenting with persistent or clinically significant pain. Further large-scale prospective studies using standardized definitions of pain, consensus-based sarcopenia criteria, consistent confounder adjustment, and repeated longitudinal assessments are required to clarify the temporal and potentially bidirectional relationship between pain and sarcopenia.

## 5 Conclusions

In conclusion, the available evidence indicates that pain is associated with higher odds of sarcopenia and sarcopenia-related outcomes in middle-aged and older adults. Positive associations were also observed for severe pain and for site-specific back or low-back pain and joint or knee pain in analyses restricted to studies using consensus-based sarcopenia criteria. However, these secondary findings should be interpreted cautiously because of the limited number of studies and the substantial between-study heterogeneity. Separate sensitivity analyses incorporating SARC-F-based studies yielded positive pooled point estimates; however, the overall-pain sensitivity analysis was substantially more heterogeneous and less stable, and screening-defined risk of sarcopenia should not be considered equivalent to sarcopenia confirmed using consensus-based criteria. Given the observational nature of the available evidence, temporal direction and causality cannot be established. These findings support heightened clinical awareness and consideration of sarcopenia screening and physical-function assessment among middle-aged and older adults presenting with persistent or clinically significant pain. Further large-scale prospective studies using standardized pain definitions, consensus-based sarcopenia criteria, consistent adjustment for confounding factors, and repeated longitudinal assessments are required to clarify the temporal and potentially bidirectional relationship between pain and sarcopenia. Interventional studies are also needed to determine whether pain management or appropriately tailored physical rehabilitation can prevent or reduce sarcopenia-related outcomes.

## Data Availability

The original contributions presented in the study are included in the article/Supplementary material, further inquiries can be directed to the corresponding author.
