# Supplementary material for: Association between pain and sarcopenia in middle-aged and older adults: a systematic review and meta-analysis
Source: Front Public Health. 2026 Aug 17;14:1891810. doi: 10.3389/fpubh.2026.1891810 (PMC13526681; doi:10.3389/fpubh.2026.1891810)
Supplement: Supplementary file 1 [file Table_1.docx]

**Supplementary Table S1**

**Search Query**

**Pubmed：**

| **No.** | **Query** | **Results** |
| --- | --- | --- |
| #1 | Pain[MeSH Terms] | 501,937 |
| #2 | Pain*[Title/Abstract] | 1,030,775 |
| #3 | Ache*[Title/Abstract] | 30,606 |
| #4 | Myalgia[Title/Abstract] | 11,072 |
| #5 | Musculoskeletal Pain[Title/Abstract] | 11,077 |
| #6 | Chronic Pain[Title/Abstract] | 64,432 |
| #7 | Arthralgia[Title/Abstract] | 9,760 |
| #8 | #1 OR #2 OR #3 OR #4 OR #5 OR #6 OR #7 | 1,230,197 |
| #9 | Sarcopenia[MeSH Terms] | 14,948 |
| #10 | Sarcopenia*[Title/Abstract] | 26,362 |
| #11 | Muscle Atrophy[Title/Abstract] | 13,743 |
| #12 | Skeletal Muscle Mass[Title/Abstract] | 10,162 |
| #13 | Low Muscle Mass[Title/Abstract] | 2,058 |
| #14 | #9 OR #10 OR #11 OR #12 OR #13 | 45,184 |
| #15 | #8 AND #14 | 1,888 |

**Web of Science：**

| **No.** | **Query** | **Results** |
| --- | --- | --- |
| #1 | TS=(**Pain*** (Topic) or **"Ache*" OR "Myalgia" OR "Musculoskeletal Pain" OR "Chronic Pain" OR "Arthralgia"** (Topic)) | [714,679](https://webofscience-clarivate-cn-s.webvpn.cdsu.edu.cn:8118/wos/woscc/summary/c64668ec-4937-4148-b7dc-a79d1e5e5297-01b37dfeda/relevance/1) |
| #2 | TS=(**Sarcopenia*** (Topic) or **"Muscle Atrophy" OR "Muscular Atrophy" OR "Skeletal Muscle Mass" OR "Low Muscle Mass"** (Topic)) | [55,758](https://webofscience-clarivate-cn-s.webvpn.cdsu.edu.cn:8118/wos/woscc/summary/81c5ee95-8bbe-4699-8a14-cfc81ad7d7ff-01b37e0346/relevance/1) |
| #3 | **#1 AND #2** | [1,810](https://webofscience-clarivate-cn-s.webvpn.cdsu.edu.cn:8118/wos/woscc/summary/59d44948-09a0-4cb1-8478-d701e3bdb0ab-01b37e07f9/relevance/1) |

**Embase：**

| **No.** | **Query** | **Results** |
| --- | --- | --- |
| #1 | 'pain'/exp | 2,114,751 |
| #2 | 'pain*':ti,ab OR 'ache*':ti,ab OR 'myalgia':ti,ab OR 'musculoskeletal pain':ti,ab OR 'chronic pain':ti,ab OR 'arthralgia':ti,ab | 1,651,523 |
| #3 | #1 OR #2 | 2,647,451 |
| #4 | 'sarcopenia'/exp | 37,632 |
| #5 | 'sarcopenia*':ti,ab OR 'muscle atrophy'/exp OR 'muscle atrophy':ti,ab OR 'muscular atrophy':ti,ab OR 'skeletal muscle mass':ti,ab OR 'low muscle mass':ti,ab | 116,911 |
| #6 | #4 OR #5 | 116,911 |
| #7 | #3 AND #6 | 11,416 |

**Cochrane Library：**

| **No.** | **Query** | **Results** |
| --- | --- | --- |
| #1 | [mh "Pain"] | 76110 |
| #2 | pain*:ti,ab,kw | 299136 |
| #3 | ache*:ti,ab,kw | 1965 |
| #4 | myalgia:ti,ab,kw | 6185 |
| #5 | "musculoskeletal pain" :ti,ab,kw | 3556 |
| #6 | "chronic pain":ti,ab,kw | 16362 |
| #7 | Arthralgia:ti,ab,kw | 6427 |
| #8 | #1OR#2OR#3OR#4 OR#5OR#6 OR#7 | 311815 |
| #9 | [mh "Sarcopenia"] | 1181 |
| #10 | sarcopenia*:ti,ab,kw | 3411 |
| #11 | "muscle atrophy":ti,ab,kw | 1258 |
| #12 | "muscular atrophy":ti,ab,kw | 1053 |
| #13 | "skeletal muscle mass":ti,ab,kw | 1631 |
| #14 | "low muscle mass":ti,ab,kw | 184 |
| #15 | #9 OR#10 OR #11 OR #12 OR#13 OR#14 | 6402 |
| #16 | #8AND#15 | 594 |

**Supplementary Figure S1**

Supplementary Figure S1. Sensitivity analyses of joint or knee pain after inclusion of CLC 2025, which assessed the risk of sarcopenia using SARC-F. (A) Random-effects meta-analysis after inclusion of CLC 2025. (B) Leave-one-out sensitivity analysis after sequential omission of each study.
